# Ion Mobility and Segregation in Seed Surfaces Subjected to Cold Plasma Treatments

**DOI:** 10.1021/acs.jafc.4c09650

**Published:** 2025-02-24

**Authors:** Alvaro Perea-Brenes, Natalia Ruiz-Pino, Francisco Yubero, Jose Luis Garcia, Agustín R. Gonzalez-Elipe, Ana Gomez-Ramirez, Antonio Prados, Carmen Lopez-Santos

**Affiliations:** † 16778 Nanotechnology on Surfaces and Plasma Laboratory, Institute of Materials Science of Seville, Consejo Superior de Investigaciones Científicas, Universidad de Sevilla, Seville 41092, Spain; ‡ Física Teórica, Departamento de Física Atómica, Molecular y Nuclear, Universidad de Sevilla, Apartado de Correos 1065, Seville 41080, Spain; § Department of Plant Biotechnology, Institute of Natural Resources and Agrobiology of Seville, Consejo Superior de Investigaciones Científicas, Seville 41012, Spain; ∥ Departamento de Física Aplicada I, Escuela Politécnica Superior, Universidad de Sevilla, Seville 41011, Spain; ⊥ Física Teórica, Departamento de Física Atómica, Molecular y Nuclear, Universidad de Sevilla, Apartado de Correos 1065, Seville 41080, Spain

**Keywords:** plasma treatment, seed germination, ion diffusion, potassium, nitrate, barley

## Abstract

Plasma treatment of seeds is an efficient procedure to accelerate germination, to improve initial stages of plant growth, and for protection against pathogen infection. Most studies relate these beneficial effects with biochemical modifications affecting the metabolism and genetic growth factors of seeds and young plants. Using barley seeds, in this work, we investigate the redistribution of ions in the seed surface upon their treatment with cold air plasmas. In addition, we investigate the effect of plasma in the lixiviation of ions through the seeds’ hull when they are immersed in water. Ion redistribution in the outer layers of air plasma-treated seeds has been experimentally determined through X-ray photoelectron spectroscopy analysis in combination with in-depth chemical profiling with gas cluster ion beams. The results show that in the shallowest layers of the seed hull (at least up to a depth of ∼100 nm) there is an enrichment of K^+^ and Ca^2+^ ions, in addition to changes in the O/C and N/C atomic ratios. These data have been confirmed by the electron microscopy/fluorescence analysis of seed cuts. Observations have been accounted for by a Monte Carlo model, simulating the electrostatic interactions that develop between the negative charge accumulated at the seed surface due to the interaction with the plasma sheath and the positive ions existing in the interior. Furthermore, it is shown that upon water immersion of plasma-treated seeds mobilized ions tend to lixiviate more efficiently than in pristine seeds. The detection of a significant concentration of NO_3_^–^ anions in the water has been attributed to a secondary reaction of nitrogen species incorporated into the seeds during plasma exposure with reactive oxygen species formed on their surface during this treatment. The implications of these findings for the improvement of the germination capacity of seeds are discussed.

## Graphical abstract

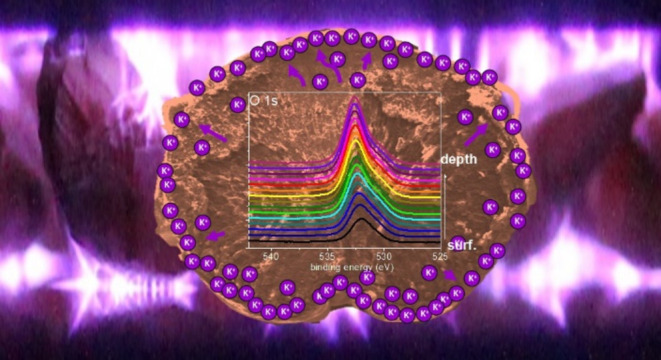

## 1 Introduction

Plasma treatment of seeds has emerged as a common procedure to improve the seedling process due to its verified contribution to increase the germination rate, reduce the contamination degree by fungi or favor the initial stages of plant growth^1–3^ through the interaction with soil microbiome,^4^ beneficial effects that are also noticeable under harsh environmental conditions.^5,6^ To account for these effects, many studies have addressed the modifications induced in the biochemistry of seeds due to the plasma treatments^7–10^ and the effect of reactive oxygen (ROS) and nitrogen (RNS) species in triggering biochemical metabolic processes that appear to modify the concentration of growth factors,^11,12^ favor the generation of enzymes,^13,14^ or even induce new gene expressions.^15,16^ However, despite the numerous papers published during the past few years on this subject, the evidence is sometimes dispersed and focused on rather specific aspects. In general, it would be desirable a more holistic approach considering aspects such as biological variability, differences in experimental conditions between laboratories, or the influence of physical processes that occur during the plasma treatment of inorganic and polymeric materials.^17–19^ In relation with the latter, there is a clear gap of information regarding the physics that develops when a plasma discharge interacts with a biological system where the mobility of ions can be affected by external electrostatic interactions and similar effects. In this context, in previous studies on quinoa,^20^ cotton,^21^ or barley^22^ seeds we have reported that K^+^ ions may segregate towards the outermost surface layer of plasma-treated seeds and that a similar diffusion process may affect other ions.^23^ Whether such segregation is beneficial for the seedlings of plasma-treated seeds is still unknown. However, the evidence that the addition of moderate amounts of hexogen potassium to plants provides favorable conditions for nitrogen metabolism, photosynthesis and, in general, nutrient assimilation and transport,^24–28^ suggests that plasma-induced ion redistribution in the interior of seeds might also play an important role for the control of specific seedling processes.

This work primarily aims to determine the effect of cold atmospheric pressure plasmas in the distribution of charged ions in the interior of barley seeds. Second, we also investigate how the plasma treatment may affect the diffusion and/or lixiviation of key ions when treated seeds are soaked in water. The found changes in the distribution of ions in a relatively thick shallow zone of the plasma-treated seeds are interpreted from a physical perspective, i.e., as a consequence of the electrostatic interactions generated when a plasma discharge contacts a solid surface. The classical theory of cold plasmas proposes that when a plasma discharge interacts with a solid surface, a boundary region around the surface, called a plasma sheath, is formed as a consequence of the higher mobility of the electrons with respect to the positively charged species in the plasma,^29,30^ and that this leads to the accumulation of negative charge on the surface of the solid.

The main assumption of the present work is that the negative charge accumulated at the seed surface during the plasma treatment may affect the distribution of ions in the seeds, attracting positively charged species (e.g., K^+^, Na^+^, Ca^2+^,···) to their surface and repelling negatively charged ones (e.g., OH^–^, PO_4_^–3^,...) from it. In a similar way than in previous attempts to model the formation of ion diffusion profiles in polymers induced by the presence of electric fields,^31^ to confer a physical basis for that hypothesis, we formulate a Monte Carlo model accounting for the migration of positively charged species towards the seed surface during plasma treatment, up to reach a canonical distribution of minimal free energy in an equilibrium state. To experimentally prove this unbalanced distribution of charged species in the interior of seeds, we analyze the chemical in-depth profiles of the outer layers of treated seeds with X-ray photoelectron spectroscopy (XPS) combined with gas cluster ion beam sputtering. This experimental approach is known to provide in-depth concentration profiles, preserving the chemical state of analyzed elements despite the etching process.^32^ In fact, the use of this technique is recommended to minimize alterations in the chemical composition of soft and sensitive materials, otherwise experiencing ion-induced reactions or element preferential sputtering.^33^ This analysis has been complemented by the scanning electron microscopy observation and the electron X-ray dispersion analysis of seed cuts.^21,22^

In the experiments, barley seeds have been subjected to a high-pressure dielectric barrier plasma discharge treatment.^6,22,34^ We confirm that the germination rate of treated seeds becomes accelerated by the plasma treatment. This result complements the evidence of an unbalanced distribution of ions in the interior of seeds and the analysis of the lixiviation of K^+^, NO_3_^–^ and other minority ions upon immersion of treated seeds in pure water. Interestingly, this study shows that surface reaction and diffusion through the seed hull may be affected by plasma treatment, confirming its importance not only to modify the biochemistry of metabolism, but also to alter physical processes that may significantly affect germination.

## 2 Materials and Methods

### 2.1 Plasma Treatment and Handling of Seeds

Barley seeds (Hordeum vulgare L.) supplied by Intermalta SA (Spain) were exposed to an atmospheric pressure air plasma treatment in a parallel plate dielectric barrier discharge reactor at room temperature. Quartz plates of 0.5 mm thickness and 10 cm diameter were placed onto two parallel stainless steel electrodes of 8 cm diameter separated by a distance of 4.2 mm. The top electrode was the active one, while the bottom electrode was grounded. A high-voltage sinusoidal signal was applied to the top electrode by a TREK (model PD05034) amplifier connected to a function generator (Stanford Research System, model DS345). A sinusoidal signal with a 1 kHz frequency and 8.6 kV voltage amplitude was set for the experiments. For these operating conditions, the current amplitude was 6.5 mA and the discharge power 5.3 W. Ambient air at a pressure of 700 mbar was fixed as plasma gas in order to get reproducible treatments (actual pressure might slightly vary depending on environmental conditions in the laboratory (see additional details in ref 22)). A set of 35 seeds was placed on the bottom electrode (grounded electrode) and exposed to air plasma for 3 min under room conditions. A rough estimation of basic plasma features like electron density or temperature was done by applying the Bolsig+ code and the electron transport equation.^35,36^

Plasma species were characterized by optical emission spectroscopy (OES) using an optical fiber connected to a monochromator (Horiba Ltd., Jobin Yvon FHR640). A diffraction grid with 1200 lines cm^–1^ and entrance and output slits of 1 mm were used to collect the spectra with a photomultiplier working in the wavelength range from 200 to 700 nm. Operating conditions were 1 nm of spectral resolution and 0.5 s integration time.

### 2.2 Germination Essay

The methodology employed for the germination essays in Petri dishes closely mirrors aspects of the industrial malting process of barley seeds. Groups of 50 seeds (pristine seeds or plasma-treated seeds) have been carefully placed on a double-layered Whatman filter paper located in 9 cm diameter Petri dishes. Four mL of Milli-Q water was employed for watering each dish. Afterward, the Petri dishes were transferred to a bacteriological incubator set (Selecta model PREBATEM 80L) and kept at 20 °C in darkness. Petri dishes were meticulously examined at intervals of 12 h, looking for signs of seed germination (penetration of the radicle through the seed coat or emergence of the coleoptile). Germinated seeds were tallied and transferred to separate Petri dishes at 12 h intervals during a 72 h cycle.

### 2.3 X-ray Photoelectron Spectroscopy Depth Profiling by Means of Gas Cluster Ion Gun Sputtering

X-ray photoelectron spectroscopy (XPS) depth profiling analysis of seeds was carried out using a Thermo Scientific “K-Alpha” apparatus at the UK’s National EPSRC XPS Users’ Service (NEXUS) at Newcastle University, UK. Control (pristine) and plasma-treated seeds at our lab in Seville were packed in plastic bags filled with nitrogen and sent to the NEXUS laboratory for testing within 24 h after plasma treatment. Three seeds of each type (plasma-treated and pristine) were stuck onto the sample holder using double-sided carbon tape. This holder was placed in a prechamber and pumped until pressure was low enough to enable the transfer to the analysis chamber. Monochromatized Al Kα radiation was used at an acceleration voltage of 12 kV and 6 mA current emission. Acquisition of survey and single core level spectra were performed with 150 and 40 eV of pass energy, respectively. The binding energy (BE) scale of the spectra has been referenced to the C 1s signal of the C–H/C–C signal at 284.5 eV. A spot size of 400 μm was used for the measurements.

Elemental depth profiles were measured at the same locations where survey and zone scans of the original samples were recorded prior to ion bombardment. The targeted depth of the profiles roughly varied from 100 to 200 nm depending on the etching process applied (see below). Two etching protocols were applied to obtain the depth profiles:

Protocol (a): depth profiling with a single charged Gas Cluster Ion Beam (GCIB) of 300 Ar atoms with an energy of 8000 eV (i.e., ∼27 eV/atom), etching time of 120 s, and estimated etching rate of 6.7 nm/cycle. Analysis time was 14 h to reach a depth of 100 nm, including 15 etching cycles.

Protocol (b): initial low current monatomic Ar^+^ sputtering with 4 keV energy for a short time (i.e., a few minutes) to remove the outermost layers of the surface material. Depth profiling with GCIB of 1000 Ar with 6000 eV (i.e., ∼6 eV/atom) and estimated etching rate of 21.6 nm/min per cycle. Analysis time was 7 h to reach a depth of 216 nm, including 10 etching cycles.

In both cases, survey and O 1s, C 1s, N 1s, Si 2p, P 2p, K 2p, and Ca 2p spectra were recorded along these depth profiling experiments. Etching rates were calibrated with a Ta_2_O_5_ standard film of a known thickness.

The fitting analysis of the C 1s and high-resolution spectra of O 1s was carried out under the assumption of a series of chemical species identified with the following binding energies: C–C (284.5 eV), C–OH (285.5 eV), C–O–C (286.5 eV), C=O (287.5 eV), and COO (288.4 eV) for the carbon region and C–O (532.7 eV), C=O (533.2 eV), and N–O (536.2 eV) for the oxygen region.^37,38^ Since the oxygen of Si–O bonds contributes to the O 1s signal, this oxygen was subtracted from the overall oxygen content to estimate the amount of oxygen associated exclusively with water or organic components. An estimation of this oxygen was made taking the Si 2p signal as a reference and a SiO_2_ stoichiometry for the Si–O compounds (note that this is a simplification because other C–Si–O bond structures can be present in the examined samples).

### 2.4 Scanning Electron Microscopy and Energy Dispersive Spectroscopy Analysis

Scanning electron microscopy (SEM) and energy dispersive spectroscopy (EDX) analyses have been done for the surface and seed cuts (i.e., cross-section) of untreated and plasma-treated barley seeds. A Hitachi S4800 SEM-FEG field emission microscope working at 2 kV has been used for morphological characterization without applying any metallization protocol. Elemental EDX maps have been obtained in an EDX-Bruker-X Flash-4010 analyzer working at 20 kV. Analyses have been done for the surface and the seed cuts (i.e., cross-section) of the barley seeds just after the plasma treatment, in comparison with the untreated seeds. Seed cuts were prepared by carefully slicing the seeds with a blade and are analyzed in the microscope within a time interval shorter than 30 min after plasma treatment.

### 2.5 Ion Release by Soaking in Water Analysis by Inductively Coupled Plasma Mass Spectrometry

To study cations (potassium, sodium, calcium, magnesium, and zinc) and anions (nitrates, chlorides, and phosphates) released in water from the plasma-treated seeds during water immersion, we have used the Agilent 7800 system from the ICP-MS Analysis Service of the Institute of Natural Resources and Agrobiology of Seville (IRNAS-CSIC). For the analysis of anions and ammonium, a segmented flow autoanalyzer, model BRAN-LUEBBE, was used during the ICP-MS test. The study was done for original seeds without any treatment and for seeds treated with plasma. A number of hundred seeds with weights ranging between 4 and 5 g were used for each experiment. Milli-Q water was used as soaking medium. The volume used for each experiment was slightly corrected considering the weight of each group (100 seeds), being on the order of 40–45 mL depending on the experiment. To check the release of ionic species, the seeds were immersed in milli-Q water for 10 and 120 min. Then, the seeds were extracted, and an aliquot of 10 mL of the liquid was taken for ICP-MS analysis. No more than 1 h elapsed since the recovery of the aliquots and their analysis by ICP-MS. To ensure a safe and uncontaminated delivery, the aliquots were stored and handled in closed tubes under dark conditions. Results are referred to the weight of seeds and will be expressed in mg/g (i.e., weight of detected species per gram of seeds).

### 2.6 Simulation Model of Ion Migration in Plasma-Treated Seeds

The diffusion of positive ions in the seed and its hull and the resulting steady-state distribution of positive ions after the plasma treatment were simulated by means of using a simplified Monte Carlo algorithm. A basic assumption of the developed model is that the seed surface becomes negatively charged as a result of the formation of a plasma sheath during exposure to the plasma.^39^ The aim of the model is to simulate the effect of the electrical field associated with this negative charge accumulated on the surface on the positively charged species (i.e., cations) distributed in the interior of seeds and thus account for the new physicochemical processes that may take place during plasma-surface interaction. It is a basic assumption of the model that at least part of the negative charge coming from the plasma will remain on the surface and will not diffuse or recombine with the positive charges already present in the system.

The model relies on a two-dimensional (2D) circular representation of the seed and on the segmentation into radially distributed cells of equal area. Positive ions are treated as point charges that can move throughout the seed. Before the treatment, the seed is electrically neutral and therefore the *N* positive ions with charge *q* are assumed to be randomly distributed inside the circular area simulating the seed. A negative density of charge, σ, compensates the charge of cations. It is assumed that this density of negative charge is uniformly distributed all over the “seed” disk. A schematic of the model is presented in the Supporting Information Figure S1. The following ratio should hold to account for the electrically neutral character of the pristine seeds in its initial state

1 $$\mathrm{Nq}+\mathrm{\sigma \pi}R^{2}=0$$

where *R* is the radius of the circle representing the seed. For the calculations, the radius has been taken to be equal to 1 mm. Since we approximate the real problem to a 2D model of the seed, its “surface” would be the perimeter of the circle, i.e., a circumference of radius *R* that bounds the interior of the seed. To simulate the effect of the plasma treatment and the accumulation of negative charge on the seed surface during plasma treatment, randomly distributed locations have been chosen to accumulate the extra negative charge associated with the formation of the plasma sheath. More specifically, we assume the accumulation of *N*_ext_ negative charges with a charge value *Q*_ext_ < 0 distributed at random positions *R⃗*_*j*_, *j* = 1,···, *N*_ext_, on the surface of the seed, i.e.,

2 $${\mathrm{R⃗}}_{j}=R\left({\cos\theta }_{j}{\mathrm{u⃗}}_{x}+\sin\theta_{j}{\mathrm{u⃗}}_{y}\right)$$

with θ_*j*_ is a random number uniformly distributed in [0, 2π]. This “extra” negative charge, created by the plasma treatment, is assumed to be fixed at the initial positions, and thus they do not move during the Monte Carlo simulation (Figure S1b in the Supporting Information).

The Monte Carlo algorithm is then run looking for the minimization of Helmholtz’s free energy *F* of the system, once the negative charges have accumulated on the surface during the plasma treatment. We recall that *F* = *U* – *TS*, where *U* is the internal energy, *T* is the temperature, and *S* is the entropy. The internal energy *U* comprises several terms stemming from all possible electrostatic interactions of the mobile positive ions in the interior of the seeds, the background negative charge homogeneously distributed in the interior of the seeds, and the fixed negative punctual charges accumulated at the surface. These immobile negative punctual charges will interact electrically among themselves and with the negative charge background density in the interior of seeds. However, from the point of view of the Monte Carlo calculations, the energy associated with this interaction is constant and does not evolve from one iteration to the next. In other words, this negative–negative charge interaction can be ignored for the simulations (the Monte Carlo scheme only considers energy differences between consecutive configurations). Therefore, calculations of the total energy are restricted to the electrostatic interaction (i) between the negative charges at the surface and the mobile *N* positive charges inside the seed, (ii) between the mobile positive charges themselves, and (iii) between the mobile positive charges and the background negative charge, as detailed below. The sum of these interactions can be formulated as

3 $$U\left(\vec{r_{1}},...,\vec{r_{N}}\right)=\sum_{i=1}^{N}U^{\left(i\right)}$$

where *U* ^(*i*)^ is the contribution of the *i*-th positive ion, characterized by its position $\vec{r_{i}}$ , to the internal energy. More specifically, *U* ^(*i*)^ can be split into three terms: (i) *U*_1_^(*i*)^, which accounts for the interaction with the fixed negative points of charge *Q*_ext_ < 0 on the surface boundary of the seed, (ii) *U*_2_^(*i*)^, which accounts for the interaction with the other mobile positive ions of positive charge *q* in the interior of the seed, and (iii)*U*_3_^(*i*)^, which accounts for the interaction of positive ions with the negative background charge upon the redistribution of the former

4 $$U^{\left(i\right)}=U_{1}^{\left(i\right)}+U_{2}^{\left(i\right)}+U_{3}^{\left(i\right)}$$

5 $$U_{1}^{\left(i\right)}=kQ_{\mathrm{ext}}q\sum_{j=1...N_{\mathrm{ext}}}\frac{1}{\left|\vec{r_{i}}-\vec{R_{j}}\right|}$$

6 $$U_{2}^{\left(i\right)}=\frac{kq^{2}}{2}\sum_{\begin{array}{l} j=1...N\\ j\neq i \end{array}}\frac{1}{\left|\vec{r_{i}}-\vec{r_{j}}\right|}$$

7 $$U_{3}^{\left(i\right)}=\mathrm{qV}\left(\vec{r_{i}}\right)$$

where *k* = (4πϵ_0_)^−1^ and *V*(*r⃗*) is the potential created by the background charge density σ, in the interior of the “seed” disk. The resulting curve of this potential is presented in Supporting Information Figure S2.

Since the experiments are done at constant (room) temperature *T*, we assume that the seeds reach the equilibrium state corresponding to this temperature. At equilibrium, the probability distribution has the canonical form, i.e., the probability density of finding the positive ions in the configuration {*r⃗*_1_, *r⃗*_2_, ···,*r⃗*_*N*_} is given by the canonical distribution

8 $$\rho \left({\mathrm{r⃗}}_{1},{\mathrm{r⃗}}_{2},...,{\mathrm{r⃗}}_{N}\right)=\frac{1}{Z}e^{-\beta U\left({\mathrm{r⃗}}_{1},{\mathrm{r⃗}}_{2},...,{\mathrm{r⃗}}_{N}\right)}$$

where β = (*k*_*B*_ *T*)^−1^, with *k*_*B*_ being Boltzmann’s constant, *T* the absolute temperature (i.e., in Kelvin), and *Z* is the partition function, which ensures the normalization of the probability density,

9 $$Z=\int d{\mathrm{r⃗}}_{1}\int d{\mathrm{r⃗}}_{2}...\int d{\mathrm{r⃗}}_{N}e^{-\beta U\left({\mathrm{r⃗}}_{1},{\mathrm{r⃗}}_{2},...,{\mathrm{r⃗}}_{N}\right)}$$

We recall that *F = −k_B_T lnZ*. In order to reach the equilibrium state, we introduce effective stochastic dynamics that drives the system to equilibrium. Details about this procedure are provided in Supporting Information S3. In the simulation, we assume that there exists an energy barrier *E*_0_ for the positive ions moving between neighboring cells, i.e., when crossing the cell boundary, a process that would have an energetic cost in a real situation. This activation energy accounts for the diffusion constraints in the seeds that hinder the movement of the positive ions. After some relaxation time, the *N* positive charges reach a final equilibrium distribution, which does not evolve (on average, there are thermal fluctuations). In an actual seed, this “final” cation distribution state resulting from the plasma treatment might be further disturbed when exposing the seeds to new conditions (e.g., immersion in water, exposure to humid environments or high temperatures, etc.).

## 3 Results and Discussion

### 3.1 Air DBD Plasma Properties and Optical Characterization

A rough estimation of the electron flux arriving at the seed surface was done using the Bolsig+ code and the electron transport equation. For this basic analysis, we disregarded possible collisional ionization and/or recombination processes that might take place within the plasma sheath at the experimental atmospheric pressure conditions of the experiment. Values of 3.74 eV, 4.8 × 10^13^ m^3^, and 8.28 × 10^–2^ m^2^ V^–1^ s^–1^ were estimated for the mean electron temperature, electron density, and electron mobility, respectively. These data allow us to calculate the flux of impinging electrons on the electrode surface and the seeds placed thereon. The obtained value was 2.87 × 10^16^ electrons m^–2^ s^–1^ under our experimental conditions.

The air plasma used for seed activation was also characterized by OES to identify some of the active species that, generated in the plasma phase, will chemically interact with the seeds. Figure 1a shows a typical OES spectrum recorded when the barley seeds were treated with atmospheric air plasma. In Figure 1b we show a picture of the interior of the reactor, with the seeds exposed to the plasma. The spectrum is dominated by the second positive system of the excited neutral molecule N_2_* [C^3^Π → B^3^Π] in the range of 290–385 nm.^40^ Notably, these species do not have an oxidative character, although they may interact chemically with the surface of the hull. It is important to stress that besides these species detected by OES, it is the most likely that other chemically active species will also form in a gas mixture of N_2_, O_2_, and other minority components. According to other works in the field,^41,42^ it is expected that nitrogen oxide molecules (NO_γ_), typically appearing in the region 225–283 nm, OH* species or O_x_* species appearing around 287–309 and 715–780 nm respectively also form in the air plasma used to activate the seeds. In our case, these species have not been efficiently detected either because their emission lines do not have enough intensity (note that seeds inside the plasma discharge produce a decrease of the light intensity collected by the fiber) or because they are so short-lived that their emission spectrum results undetectable. We should mention in this regard that in a previous work on barley seeds, we showed that ROS species resulting from the interaction with plasma species of oxygen accumulate in the seeds as a result of the plasma treatment. The chemical effect of these ROS could be emulated by hydrogen peroxide, suggesting that they are diatomic species of oxygen, likely peroxide or superoxide, both with a significantly high oxidative character.^22^

**Figure 1:**
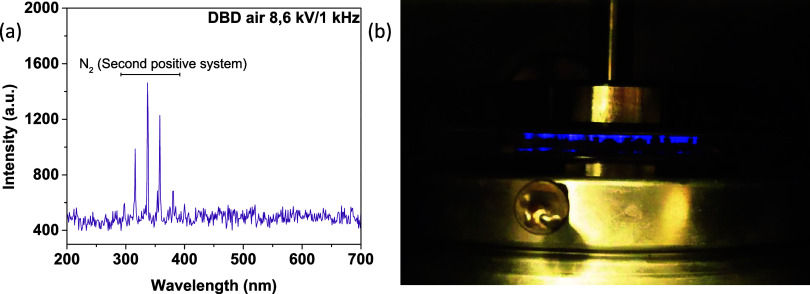
(a) Optical emission spectra corresponding to the air plasma discharge in the presence of barley seeds. (b) Picture of the air plasma treatment on barley seeds.

### 3.2 Germination Rate of Plasma-Treated Barley Seeds

The plasma-treated barley seeds behave in a similar way to those in previous experiments carried out with this type of seed.^6,22^ In agreement with the already reported results, plasma-treated seeds experienced an acceleration in the germination rate, as illustrated in Figure 2. This acceleration is particularly noticeable after the first 18 h, where the number of germination events was 25% higher for the plasma-treated with respect to pristine control seeds. In previous works, we have verified that in addition to this increase in the germination rate there were other changes in the phenotype of young plants when the seeds were sown in soil. These changes, both in germination rate and in the seedling process have been widely studied and commonly attributed to the chemical interaction of reactive oxygen species (ROS) and reactive oxygen and nitrogen species (RONS) accumulated in the seeds during the plasma–seed interaction with the normal metabolic chains of seeds and young plants.^43,44^ Unlike this common approach, herein we focus attention on the changes in the distribution of ions in the interior of seeds. This analysis has been carried out by XPS complemented with SEM-EDX analysis of cross-section cuts of the seeds.

**Figure 2:**
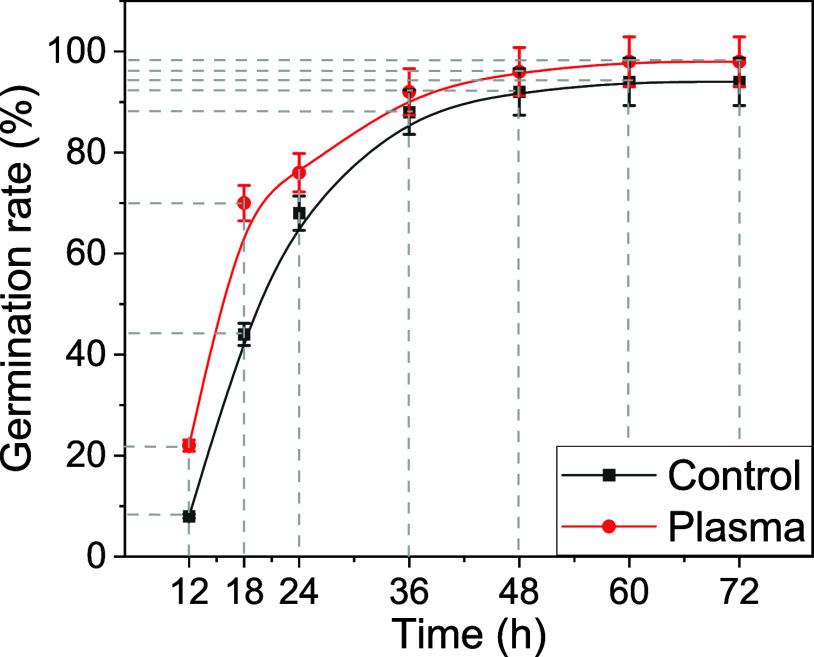
Germination rate in percentages for pristine (control) and plasma-treated barley seeds as a function of time since they were put in the Petri dishes.

### 3.3 In-Depth Chemical Analysis of Plasma-Treated Seeds

The plasma treatment herein applied is similar to that used in previous studies on barley seeds, and, as expected, it leads to similar beneficial effects on seed germination and plant growth.^6,22^ In this work, we specifically investigated the changes in surface composition that are produced by the plasma treatments. The gentle plasma conditions used to treat the seeds did not induce damage signs on the seed surface morphology, as can be appreciated in the SEM/EDX analysis reported in Figure 3 and in the Supporting Information S4. It is noticeable that the SEM images of seed cuts included in these two sets of figures reproduce well the main morphological features of the inner structure of Barley seeds previously reported by Geng et al. and that this internal morphology is not affected by the plasma treatment.^45^ However, comparison of the EDX maps of the seed cuts in Figure 3 taken before and after plasma treatment show a certain hull enrichment of elements such as K (see the more intense color of hull zone in the K distribution zoomed map after plasma treatment). The thickness of this enrichment zone could be estimated to be several tens of microns (∼40 μm^46^). By this analysis, it is also important to highlight that SEM/EDX reveals the presence of silicon nodules at the surface of barley seeds.^22^ This observation will be taken into consideration when analyzing the O 1s photoelectron spectra recorded during the depth profiling analysis by XPS. Additional elements such as Mg, Na, Cl, Al, Ca, and S have been detected on both treated and untreated seed cuts (see the Supporting Information, Figure S5) although without clear evidence of accumulation or depletion at the hull region. It is important to remark that the compositional distributions presented in Figure 3 did not significantly vary upon storage under ambient conditions for some days after the plasma treatment. This supports the idea that the accumulation of potassium on the external region of the seed hull must proceed through diffusion from the inner regions of the seed.

**Figure 3:**
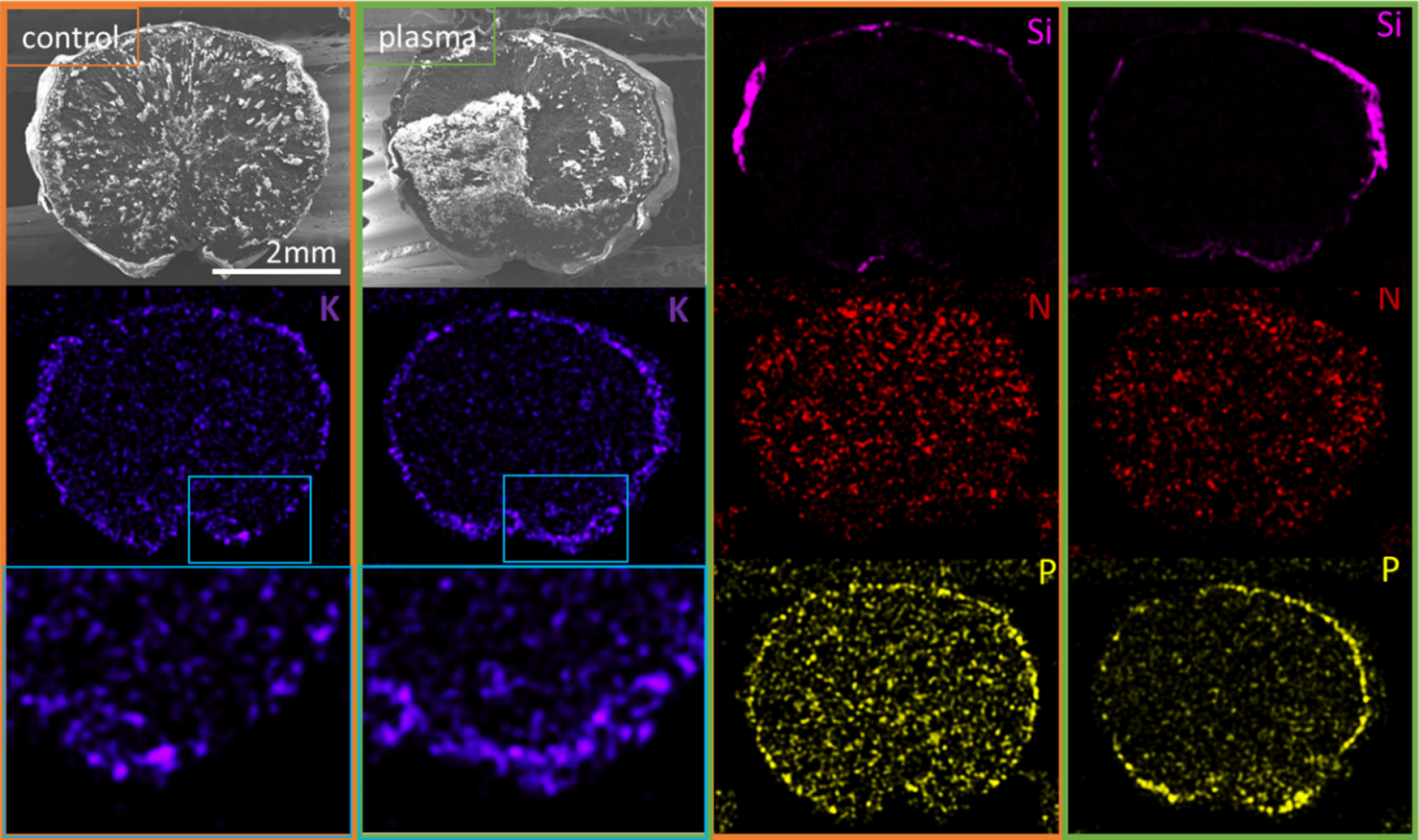
SEM-EDX analysis (K, Si, N, P maps, including a couple of enlarged zones of the K maps) of cross-sectional cuts of the seeds showing the enrichment of some minority elements in the outer zones of hull after the plasma treatment: (orange squares) control barley seed and (green squares) barley seed exposed to air plasma treatment for 3 min.

XPS provides a more precise analysis of the chemical composition of the most external regions of the seed surface. Table 1 summarizes the element concentration in the outermost outer layers monitored by this technique for the pristine and plasma-treated seeds before depth profiling. The probe depth with a conventional XPS analysis can be roughly estimated in 1–2 nm thickness (i.e., a very thin surface region). It is apparent in this table that the surface composition of this outer layer drastically changes after plasma treatment. Similar changes in XPS compositions have been previously reported for barley, cotton, and quinoa seeds subjected to plasma treatments.^20–22^ The common tendencies observed when comparing atomic concentrations can be summarized as follows: plasma treatment induces a decrease in the carbon and an increase in oxygen and, for the barley surface, also in silicon. It is also noticeable the increase in the percentages of minority elements such as K, Ca, and N after plasma treatment. The increase in oxygen and the decrease in carbon are a direct effect of the interaction of carbon-containing materials with oxygen-containing plasmas and are the result of the partial oxidation of organic matter and the formation of –C=O, –C–OH, or −COOH species.^47,48^ It is also likely that complete oxidation may also affect the organic matter in the outermost atomic layers of seeds, leading to the formation of CO_2_ and H_2_O. Such a series of effects have been widely reported for polymer materials and, as mentioned before, also for seeds. At the surface of barley seeds, Figures 3 and S4 in the Supporting Information show the presence of a relatively large number of spotted SiO_2_ agglomerates, rendering a significant average concentration for Si at the surface, as monitored here by XPS. The increase in the Si concentration after plasma treatment is likely the result of the cleaning removal of adsorbed airborne hydrocarbons or other carbon contamination on the surface of seeds, as well as the possible removal of an organic layer covering these agglomerates. We assume that these SiO_2_ agglomerates are inert and do not play any significant role in the ion redistribution processes that are the subject of the present study. Therefore, regarding plasma reactivity and the possible effect on seed germination, the most significant feature is the increase in K, Ca, and N concentrations found for the plasma-treated seeds.

**Table 1:** Percentage of Elements Determined by XPS in the Pristine and Plasma-Treated Seeds

	atomic percentage (%)	
	control seed	plasma-treated seed
C	88.5	52.8
O	9.2	37.6
Si	0.4	4.8
N	0.8	2.1
K	0.6	1.7
P	0.2	0.2
Ca	0.3	0.8

Since conventional XPS only provides information over the outermost surface layer of samples (i.e., approximately 1–2 nm of thickness) it is important to determine whether these or similar changes also affect the inner layer of the seeds. With this purpose, a series of depth profiling experiments were carried out using the two experimental protocols described in the Experimental section. Cluster ion depth profiling according to protocol 1 permits monitoring the evolution of element concentration along a layer of ca. 100 nm thickness. Figure 4 shows a series of depth profiles recorded for a reference and a plasma-treated seed. Depth profiles are plotted as a function of the GCIB etching time. Data are reported for the majority elements C, O, and Si in Figure 4a,b. The depth profile recorded for the control seed reveals an enrichment in carbon in the outermost layers of seeds (some contribution by airborne carbon contamination cannot be discarded) and a progressive and smooth enrichment in O together with a relative depletion in C at deeper zones of the seed. However, since the Si concentration also increases with depth, part of this increase in oxygen should be associated with the SiO_2_ agglomerates present at the seed surface. In practice, discounting the percentages of silicon and associated O, it appears that carbon concentration is rather constant all along the etched thickness, with average values around 55%. This evolution of C concentration agrees with the known structure and the thickness of several microns of seed hull, which is known to be mainly formed by cellulose, tannins, and small amounts of some polyphenols, i.e., compounds very rich in carbon.^49^

**Figure 4:**
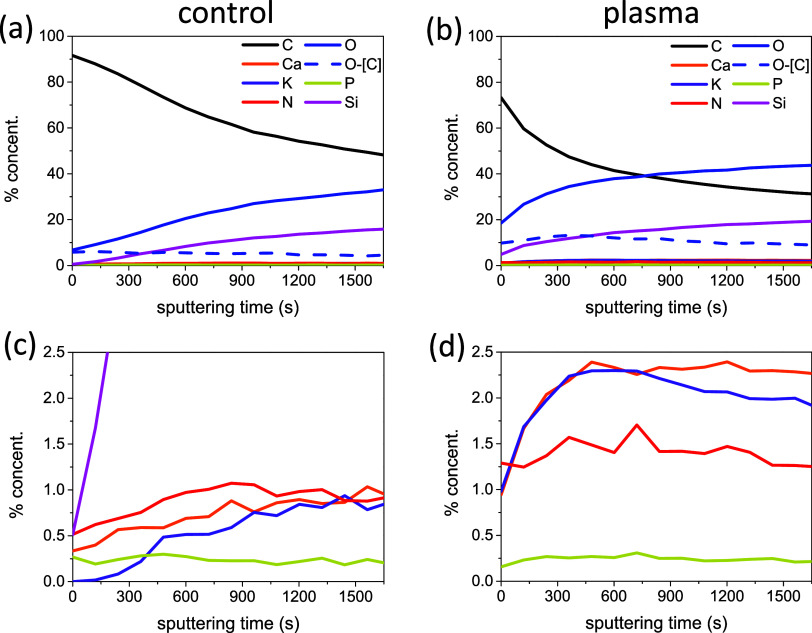
Element depth profiles for the control (a, c) and plasma-treated (b, d) seeds (up to 100 nm in depth). Dashed lines represent the depth profile of oxygen associated with the organic component of the outer layers of seeds (i.e., once discounting the oxygen associated with silicon).

This situation drastically changed for the seed exposed to the plasma. The depth profiles in Figure 4b indicate that once discounting the oxygen associated with silicon, there is still a non-negligible amount of oxygen with values up to 11.5% that distributes all along the depth of the etching profile. This renders an O/C ratio varying from 0.13 at the surface to 0.28 after etching with 100 nm of thickness. In other words, a considerable amount of oxygen has become incorporated into the outer layers of the seed hull, extending along more than 100 nm. The etching profiles obtained using protocol 2 (see Supporting Information S6), confirm that oxygen becomes incorporated in the carbon-rich matrix of the seed hull and that this enrichment in oxygen occurs at least up to a thickness of 216 nm. The oxygen incorporation into organic materials upon plasma exposure is a common feature for polymers and related materials.^50,51^

However, the most interesting issue in relation to the main hypothesis of this work, i.e., that charged ions may migrate toward the surface upon plasma exposure, is the analysis of the minority elements also recorded during the depth profiling analysis. Depth profiles of minority elements K, Na, Ca, N, and P are reported in Figure 4c,d for the original and plasma-treated seed, respectively. Most significantly, the concentrations of K, Ca, and N along the whole depth profile are higher in the plasma treated than in the control seed, indicating a net enrichment in these elements after the plasma treatment. Moreover, the profiles for K and Ca differ in shape with respect to those in the control seed, where the concentration profiles vary smoothly. We propose that the detected enrichment in K and Ca in the outermost layers of the seed hull (i.e., at least up to 100 nm in this experiment) results from electrostatic interactions developed between the negative charges accumulated on the surface of the seeds during the plasma treatment and the relatively free and mobile positive ions (K^+^, Ca^2+^, Na^+^) existing in the interior of seeds. Besides Ca and K, another minority element whose distribution increased in this experiment along the examined thickness of seeds was nitrogen. Meanwhile, the P distribution remained almost unaltered after the plasma treatment.

To properly explain the observed changes in the concentration of the detected elements at the outermost surface layers of the plasma-treated seeds, we deemed necessary to gain a deeper understanding of the chemical nature of the species detected by XPS. Figure 5 shows selected C 1s, O 1s, and N 1s spectra taken along the depth profiles in Figure 4 both for the reference and plasma-treated seeds. Equivalent series of spectra for the Si 2p, K 2p, Ca 2p, and P 2p levels are reported as Supporting Information Figure S7. The binding energy values assigned to the main functional groups of the elements detected at the barley surface before and after exposure to the plasma discharge are gathered in Table S8 in the Supporting Information. Particularly, the silicon contribution detected at the surface of the plasma-treated seed is located at 102.8 eV of binding energy, a value pointing to the presence of Si–C–O groups. This result agrees with previous works quantifying the silicon content of different varieties of barley seeds.^52,53^ After etching, the peak shifts to 103.4 eV, a BE value typical of SiO_2_.^54^ The appearance of doublet peaks around 292.8 and 295.6 eV^55^ and 346.7 and 350.2 eV^56^ are an indication of the presence of K^+^ and Ca^2+^, respectively. The shape of the spectra/BE of 2p_3/2_ peaks do not change along the depth profile, supporting that the same chemical species appear distributed along the whole analyzed thickness of the plasma-treated barley seed. Otherwise, the photopeak around 132.7 eV is commonly assigned to the P–O functionalities, likely due to phosphate anions.^57^

**Figure 5:**
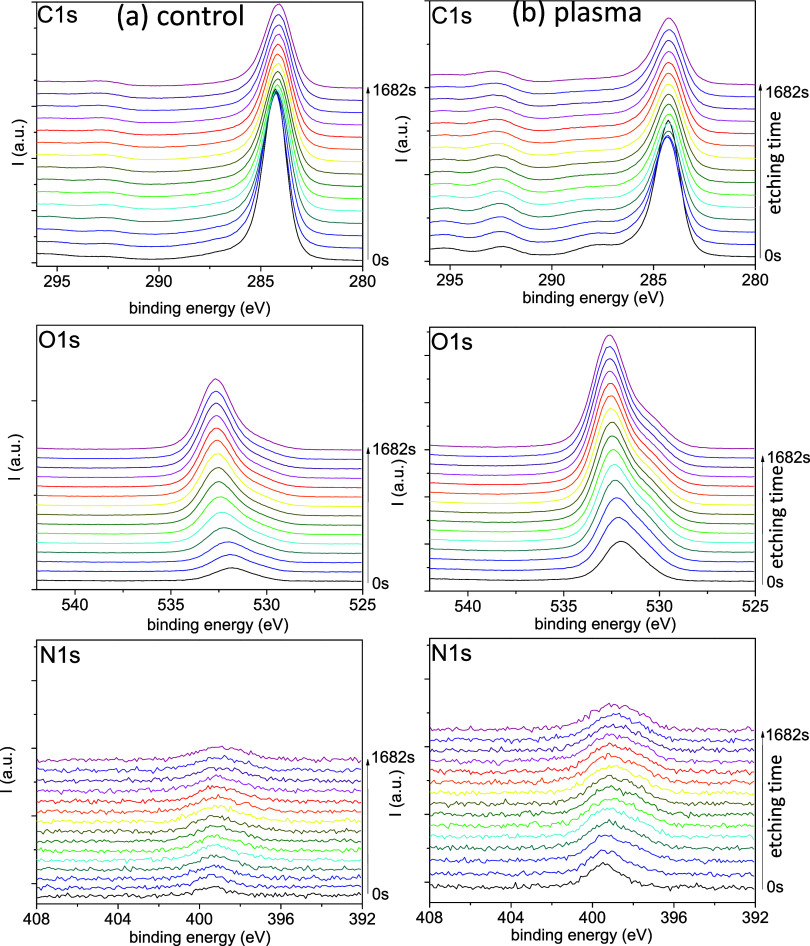
Selected C 1s, O 1s, and N 1s spectra taken along the depth profiles (i.e., progressive etching times from 0 to 1682 s as indicated with the arrows) for control (a) and plasma-treated (b) barley seeds.

The series of spectra in Figure 5 indicate a progressive variation of the O 1s, C 1s, and N 1s peaks with the etching time. These changes occur in parallel with the quantitative variations in atomic percentages reported in Figure 4. To get more precise information on the chemical characteristics of the chemical bonds present in the examined layers of the seeds and how they vary with the etching time, a fitting analysis was carried out for selected spectra taken from Figure 5. Fitting of the C 1s spectra was carried out under the assumption of the contribution of the following functional groups: C–C (284.5 eV), C–OH (285.5 eV), C–O–C (286.5 eV), C=O (287.5 eV), and COO (288.4 eV)^37,38,58^ (see Figure 6). We must note that the contribution of C–N functional groups will overlap in the region around 286.2 eV and has not been explicitly considered to simplify the fitting analysis because it would be indistinguishable from the C–O contribution.^59^ The C=O and COO functional groups were relatively more intense at the surface of the plasma-treated seeds (see the enlarged representations of the spectra at 0 s etching time in Figure 6c,d). This can be taken as a hint of plasma etching oxidation of the outer carbonaceous layers of the seeds. Significantly, Figure 6c also shows that the concentration of these groups decreases in the deeper layers of the plasma-treated seeds that are examined after 1600 s of etching time. In addition, since these deeper zones of the seeds are not directly exposed to the plasma, we assume that the small relative increase in C=O and COO functional groups in the plasma-treated seeds that can be deduced when comparing the 1600 s spectra in Figure 6c,d must proceed from a certain rearrangement mechanism of carbon functional groups and/or the diffusion of reactive species of oxygen from the surface toward the interior of seeds.

**Figure 6:**
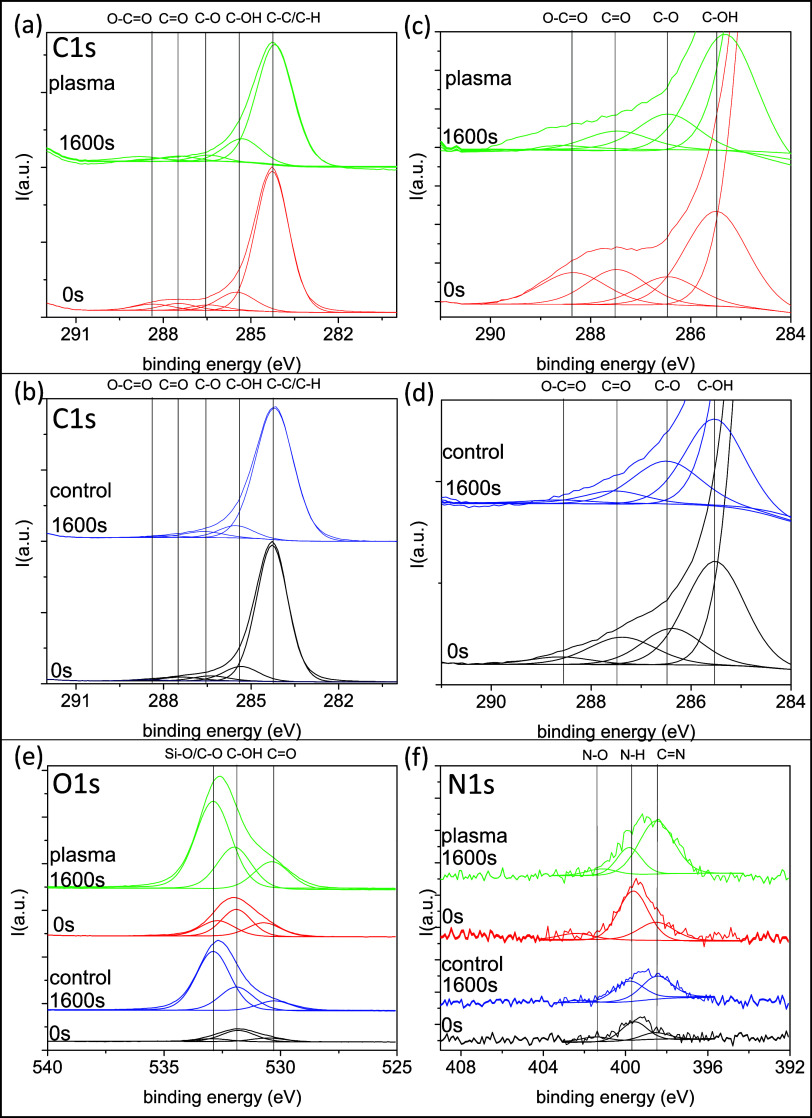
Fitting analysis of selected spectra taken from Figure 5 corresponding to the first (i.e., 0 s) and last (i.e., 1682 s) spectra of the series, both for the control and plasma-treated seeds: (a, b) C 1s regions; (c, d) zoomed C 1s regions; (e) O 1s regions; and (f) N 1s regions. Spectral intensities have been normalized to the C 1s control 0 s area.

The O 1s spectra (c.f, Figure 6e), can be fitted with a series of bands that can be attributed to O–Si/O–C (at 532.8 eV), HO–C (532.0 eV), and O=C (530.8 eV).^60,61^ The intensity of the O 1s band attributed to O–Si bonds has been estimated from the measured intensity of the Si 2p peak. The most significant difference between control and plasma-treated seeds is the increase in the intensity of the band at around 530 eV due to surface oxidation of carbon. Interestingly, the contribution at around 532.8 eV, which is compatible with O–Si bonds, and contributions at higher BEs might be also due to some form of diatomic oxygen of peroxide or superoxide type.^62^ These chemical species are usually taken as an expression of ROS, which have been demonstrated to be very active in triggering metabolic reactions beneficial for the germination and seedling of seeds.^63^

The N 1s spectra (c.f., Figure 6f) have been fitted under the assumption of three bands attributed to N=C (398.5 eV), C–N–H_*x*_ (amine at 399.2 eV) and protonated amine (C.N–H_*x*+1_^+^) or N–C=O (amide) groups (401.3 eV).^64^ By this analysis, it is relevant to highlight that there are no clear contributions at BEs higher than 402 eV, a region where nitrogen–oxygen bonds appear in the N 1s photoelectron spectra (NO_*x*_ or similar species are characterized by BEs as high as 405 eV). Therefore, this analysis discards the existence of significant concentrations of RONS species on the surface of the seeds even after the plasma treatment. We assume that the observed increase in nitrogen (c.f. Table 1 and Figure 4) is due to nitrogen species bonded to carbon very likely through direct interaction of the carbon-rich structure of the hull with excited nitrogen species of plasma such as those detected by OES (see Figure 1). It is noteworthy that the found increase in nitrogen concentration extends through at least a thickness of 200 nm of seeds (see Supporting Information S6), thus indicating that the chemical effects of plasma does not remain confined at the outermost surface layer but extends to the interior of seeds. Interestingly, this effect is less pronounced for oxygen linked to carbon, whose concentration, within the incertitude limits imposed by the presence of SiO_2_/Si–C–O agglomerates, only starts to decrease after the first 80 nm of thickness (see Figures 4 and S6).

### 3.4 Simulation of Positive Ion Redistribution During Plasma–Seed Interaction

The Monte Carlo model described in the Experimental section and Supporting Information S3 has been applied to account for the redistribution of positive charges between the inner and outer layers of the seeds upon plasma activation. This simulation relies on the hypothesis that an extra negative charge accumulates on the surface of seeds during this process and that the developed electrostatic interactions are responsible for the observed surface enrichment in K^+^ and Ca^2+^ ions detected by XPS. The application of the Monte Carlo algorithm permits following the evolution in the distribution of positive charge from its initial homogeneous state prior to plasma exposure to a final equilibrium state of minimum free energy. Typical configurations for the initial and final states of the system are shown in the schematics in Figure 7a. The developed Monte Carlo algorithm was computed using the following parameter values

$$N=10^{3}\;\mathrm{ions},N_{\mathrm{ext}}=10,Q_{\mathrm{ext}}=4{\times 10}^{5}q,R=1\mathrm{mm},T=300\;K,\mathrm{and}{,E}_{0}=4k_{B}T$$

**Figure 7:**
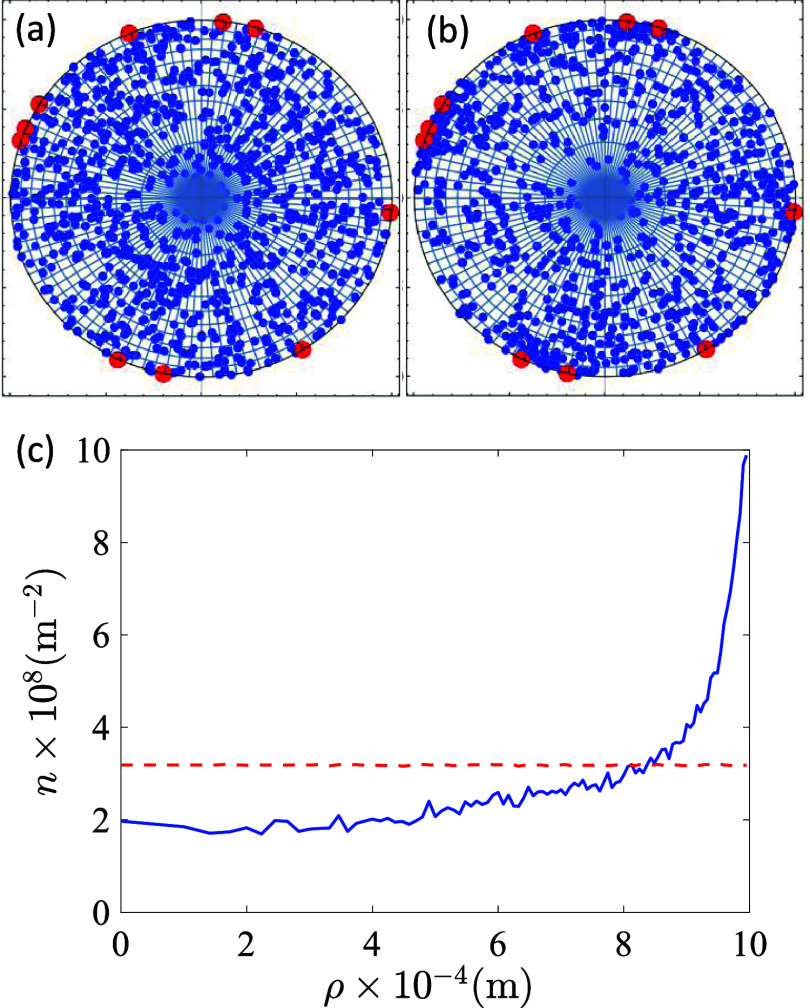
Typical configurations of the ions in the initial (a) and final (b) stages of Monte Carlo simulation. Specifically, the mobile positive ions (blue) and the quenched external negative charges (red) are plotted, at the beginning (a) and the end (b) of the iteration process. Initially, the positive ions are randomly distributed in the circle, whereas the external negative charge accumulates at random locations in the shell. In the final state, an extra concentration of positive ions appears in the cells close to the negative charges. (c) Concentration of the positive charge of cations as a function of the distance to the center of the circle ρ in the equilibrium state. The solid horizontal straight line represents the theoretical concentration of a uniformly distributed set of positive ions (i.e., in the initial state).

These values do not reproduce the actual conditions of the experiment but aim to provide a reference frame of the physical processes occurring in the seed due to the plasma treatment. Neither the model size (i.e., R) nor the amount of accumulated charge (i.e., *N*_ext_ *× Q*_ext_) are in direct correlation with the actual size of a barley seed or the total amount of negative charge impinging on the seed surface during the plasma treatment time that, taking into account the estimated flux of electrons, would be in the order of 3 × 10^16^ electrons m^–2^. Thus, although the values selected for *N*_ext_ and *Q*_ext_ have been chosen arbitrarily, they comply with the condition that, in absolute terms, *N*_ext_ *Q*_ext_ *≫ Nq*. In other words, the model assumes that during the plasma treatment the extra negative charge accumulated on the seed surface due to the higher mobility of electrons, compared to that of the positively charged species in the plasma, is much bigger than the overall charge of the mobile positive ions existing in the interior of the seed. This condition ensures that electrostatic interactions developed during the plasma treatment are the main factor inducing the migration process of the cations toward the surface.

The value selected for the energy barrier corresponds to a potential *V*_0_ = *E*_0_/*q* equal to 0.1034 V, i.e., a value within the same order of magnitude than the resting membrane potential in plants.^65^ An additional remark should be made with respect to the *N*_ext_ spots at the surface that concentrate all negative charge *Q*_ext_. They defined in the model the distribution of negative charge accumulated on the shell of the seed during the plasma treatment. In real seeds, the shell does not have a perfectly smooth morphology but has “cusps” at which the charge would be preferentially deposited because of the well-known sharp-point focusing effect on the electric field. This justifies why the charges *Q*_ext_ accumulate in the model at *N*_ext_ random points along the perimeter of the circle, as defined by eq 2. In our simple model, to lower the computed number of interactions and to accelerate the convergence of the algorithm, we have considered a limited number of charged points (i.e., *N*_ext_ = 10) each one with a “high” accumulation of negative charge, i.e., *Q*_ext_ ≫ *q*.

Once the system has reached its equilibrium state in the Monte Carlo simulations, the positive charges representing the cations in the seed appear to undergo a redistribution consisting of preferential accumulation in the outer cells of the circle in the vicinity of the *N*_ext_. This can be clearly appreciated in Figure 7b,c, the latter showing the dependence of the concentration of positive charge on the radial coordinate ρ (see details of this calculation in Supporting Information S9). This plot clearly shows that the positive charge of seed cations becomes concentrated near the shell (i.e., where depth profiles revealed a maximum concentration of K^+^ and other positive cations) due to the electrostatic attraction exerted by the external negative charge during the plasma treatment. It is convinient in this regard to insist on the difference of probing depth provided by XPS depth profiling (i.e., 100–200 nm) and the results of calculations in Figure 7c, where the enrichment of positive ions extends to several hundred microns depth. This thickness is of the same order of magnitude as the K^+^ enrichment zone determined by EDX analysis (c.f. Figure 3), thus supporting the validity of our Monte Carlo simulations, at least at a qualitative level.

The evaluation of the variation of the system energy (i.e., U) from the initial state (c.f. Figure 7a) to the final state (c.f. Figure 7b) rendered a change from approximately −1.25 × 10^–20^ J/ion to ca. −1.45 × 10^–20^ J/ion. Convergence was reached after 6 × 10^5^ iterations (the evolution of the computed energy of the system along the iterative convergence process can be seen in Supporting Information S9).

It is noteworthy that simulation results similar to the ones presented here were obtained by substituting the homogeneous distribution of negative charge density σ within the circle by *N* negative point charges distributed uniformly along the cells (i.e., mimicking the distribution of positive charges in the initial state). This supports the validity of the model assumptions regarding the compensation of positive charge in the interior of the seeds. It must also be stressed that the actual values of the parameters used for the calculations (i.e., values of *N*, *N*_ext_, *Q*_ext_,...) affected the degree of the observed effects but not their general, qualitative, trend. For example, the incorporation of less negative charge on the surface gave rise to a lesser accumulation of positive ions at the outer layers of the disk. Similarly, a smaller potential barrier between cells made that the equilibrium state was reached after fewer iterations. However, the pervasive common result was that the external negative charge incorporated on the surface during plasma treatment acts as a thrust to redistribute the positive ions around them, i.e., at the outer layers of the system as observed by XPS and EDX in the experiments.

### 3.5 Analysis of Water Ion Release Processes

The surface analysis of the seed hull before and after plasma treatment has revealed the occurrence of significant chemical and ion redistribution changes in a thickness of at least 200 nm (the actual thickness is not accessible by the GCIB-XPS experiment but can be estimated to be a few micrometers as suggested by the EDX potassium maps in Figure 3). The experiments in this section aim at determining whether the degree of ions released into water is affected by the plasma treatment or not. Experiments have monitored the release of ions from the seeds when they are immersed in pure water. Figure 8 shows the amount of cations such as K^+^, Na^+^, NH_4_^+^, and Mg^+^ determined by ICP-MS in distilled water after the immersion of pristine and plasma-treated seeds for 10 and 120 min (see the specific conditions in Section 2). It is apparent that except for NH_4_^+^, the release of cations, particularly K^+^ and Na^+^, is always higher from the plasma-treated than from the control seeds. This result suggests that the protection and isolation functions of the hull membrane have been somehow weakened for the plasma-treated seeds. We can also postulate that the enrichment of cations at the outer layers of the seed as an effect of the plasma treatment (c.f., Figures 4–6) makes more favorable their release to the water upon immersion.^66,67^

**Figure 8:**
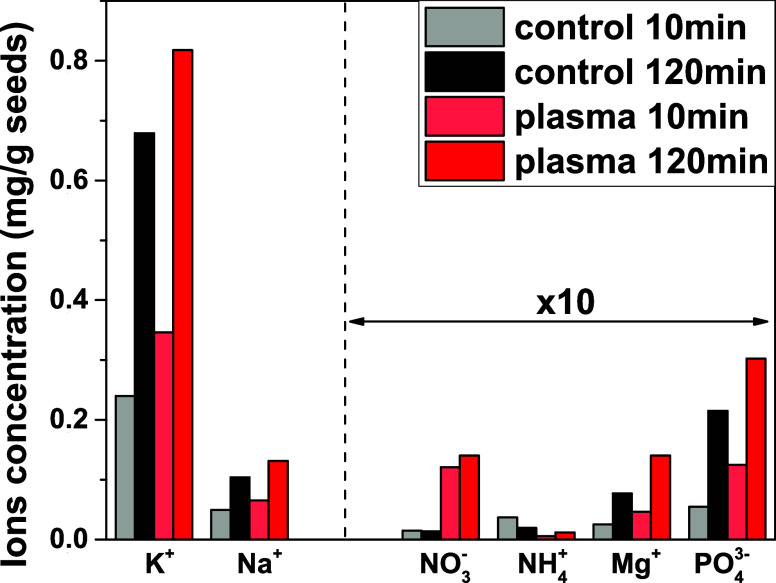
ICP-MS analysis of ions (mg/g) released from control and plasma-treated seeds immersed in distilled water for 10 and 120 min. The concentrations of NO_3_^–^, NH_4_^+^, Mg^2+^, and PO_4_^3–^ ions are multiplied by a factor of 10 to make them observable.

Interestingly, the very little lixiviation found for anions, particularly PO_4_^3–^, is also enhanced for the plasma-treated seeds. The case of NO_3_^–^ is particularly relevant in this regard. First, because practically no NO_3_^–^ ions were lixiviated for the control seeds (this agrees with that a little or negligible amount of free NO_3_^–^ exists in the interior of barley seeds^68^), while a net amount was detected in the plasma-treated seeds. Second, because in the plasma-treated seed, the amount of NO_3_^–^ was rather similar after 10 or 120 min of seed immersion in water. The detection of lixiviated NO_3_^–^ from the plasma-treated seeds is somehow contradictory with the fact that no NO_*x*_ species were detected by XPS on the surface of plasma-treated seeds (c.f. Figure 6), while other forms of nitrogen significantly increased with respect to the control seeds. We hypothesize that the detected nitrate forms through a chemical reaction involving the extra accumulated nitrogen in the outer layer of plasma-treated seeds and the ROS species formed and adsorbed on the seeds during the air plasma treatment. The process could be described by the following reaction schematics, which would take place in the presence of water and lead to the formation of NO_3_^–^ and its release to the water where the seeds are soaked:

10 $$C-N\left(H\right)+\mathrm{ROS}*\to {{\mathrm{NO}}_{3}}^{-}\left(H_{2}O\right)$$

The results disclosed in this work identified some of the physical effects that may take place when seeds are exposed to plasma. Unlike most contributions in this area have been focused on chemical, biochemical or even gene expression effects of the plasma treatment,^1,3,5^ the evidence gathered in this investigation has shown that physical interactions are not negligible and may induce the redistribution of ions in the interior of seeds. Whether such effects can be linked with the known chemical and biochemical factors affecting seed and young plant metabolism is a question to be addressed in specific works on this topic. Interestingly, the current paradigm to account for these biochemical or gene expression effects relies on the effect of ROS and/or RONS in triggering specific metabolic reactions contributing to increasing the germination rate of seeds or improving the seedling process.^11,43,63^ We hypothesize that the detection of NO_3_^–^ anions in the water after soaking the plasma-treated seeds is a hint of a water promoted-reaction between adsorbed ROS and the nitrogen species incorporated in the outer layers of the seeds. Nitrate species are known as precursors of the formation of amino acid in plants through its enzymatic reduction to nitrite and ammonia species. Also, as a signaling species responsible for reducing the dormancy of seeds and triggering the germination processes.^69,70^ In this regard, the formation of NO_3_^–^ through reaction 10 would be an adjuvant factor of the germination. The redistribution of K^+^ and other cations inside the seed might be another one. Different works have reported that exogen K^+^ may contribute to improving the seed germination and the seedling process.^71,72^ However, at present, the implications of these ion-linked processes for the control of seed metabolism and/or for triggering specific biochemical growing factors are questions open for debate that require specific new works directly addressing these open questions.

## 4 Conclusions

The results of this work have clearly established that exposing seeds to cold atmospheric air plasma produces changes in the distribution of charged species in their interior. Specifically, it has been proved that positive ions diffuse and tend to accumulate over the outer layers of seeds in a thickness of at least 200 nm and that likely extend up to some microns. The driving force for this redistribution has been associated with the accumulation onto the seed surface of negative charge, a process naturally occurring whenever a body is exposed to plasma and a plasma sheath forms around it. The simulation of the cation diffusion process with a Monte Carlo model has given support to the experimental results and opened the way to using simple physical models to describe complex mechanisms occurring in living structures. In addition to a redistribution of positive charge, the XPS analysis of the composition of the outer layer of the seeds exposed to the plasma has proven the occurrence of important chemical modifications that extend to thicknesses of, at least, several hundred nanometers. As a hypothesis, we propose that in the presence of water, the extra nitrogen incorporated in these outer layers in the form of C–N or C–NH_*x*_ species might react with ROS species to yield NO_3_^–^, a known signaling molecule known for triggering germination and reducing dormancy in seeds. This study about the redistribution of ions in plasma-treated seeds might be linked in the future with other changes in metabolic functions contributing to better understanding, controlling, and improving the seedling (germination) processes.

## Supplementary Material



## Notes

Scheme for the distribution of positive charges inside the seed before the plasma treatment and effect of plasma exposure (S1); Calculation of the electric potential due to the homogeneous distribution of negative charge in the interior of the seed (S2); onte Carlo principles enabling that the system reaches a final equilibrium state (S3); SEM and EDX analysis of barley seeds (S4); Additional EDX analysis of barley seeds cuts (S5); Etching profiles according to protocol 2 for barley seeds before and after being subjected to plasma treatment (S6); XPS spectra of minority elements recorded after GCIB depth profiling of pristine and plasma-treated barley seeds (S7); Summary of the binding energies and atomic concentration of the functional groups at the barley surface before and after the plasma treatment obtained by fitting the XPS spectra (S8); Evolution of the internal energy per ion (S9); Calculation of the distribution of positive charge in the final state of the system (S10) (PDF)
