# Supplementary material for: Ion Mobility and Segregation in Seed Surfaces Subjected to Cold Plasma Treatments
Source: J Agric Food Chem. 2025 Feb 24;73(11):6486–99. doi: 10.1021/acs.jafc.4c09650 (PMC12135037; doi:10.1021/acs.jafc.4c09650)
Supplement: Supplementary file 1 [file jf4c09650_si_001.pdf]

Supporting information

Ion mobility and segregation in seed surfaces subjected to cold plasma treatments

Alvaro Perea-Brenes,[a] Natalia Ruiz-Pino,[b] Francisco Yubero,[a] Jose Luis Garcia,[c] Agustín R. Gonzalez-Elipe,[a] Ana Gomez-Ramirez,[a,b] Antonio Prados,[b*] Carmen Lopez-Santos[a,d*]

[a] Nanotechnology on Surfaces and Plasma Laboratory, Institute of Materials Science of Seville, Consejo Superior de Investigaciones Científicas-Universidad de Sevilla, Seville 41092, Spain

[b] Física Teórica, Departamento de Física Atómica, Molecular y Nuclear, Universidad de Sevilla, Apartado de Correos 1065, Seville 41080, Spain

[c] Department of Plant Biotechnology, Institute of Natural Resources and Agrobiology of Seville, Consejo Superior de Investigaciones Científicas, Seville 41012, Spain

[d]Departamento de Física Aplicada I, Escuela Politécnica Superior, Universidad de Sevilla, Seville 41011, Spain,

*mclopez@icmse.csic.es; prados@us.es

Supporting information S1.- Scheme for the distribution of positive charges inside the seed before the plasma treatment and effect of plasma exposure.

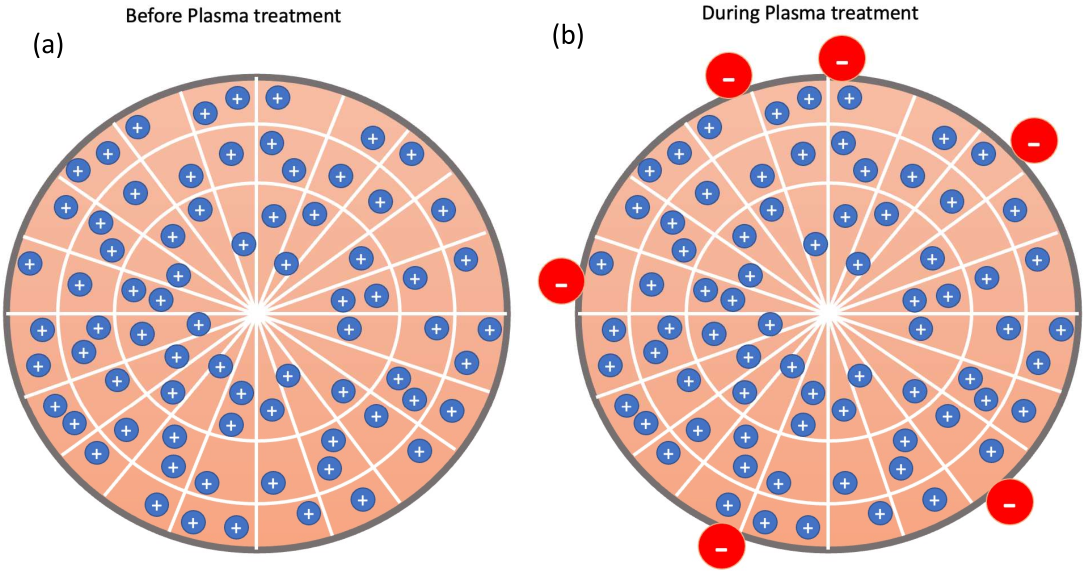

Fig. S1: Theoretical model of the ions distribution in the seed both before (a) and during (b) the plasma treatment. During the plasma treatment, specific locations at the seed surface become negatively charged. The configuration shown in (b) constitutes the initial state for the Monte Carlo simulation.

Supporting information S2.- Calculation of the electric potential due to the homogeneous distribution of negative charge in the interior of seed

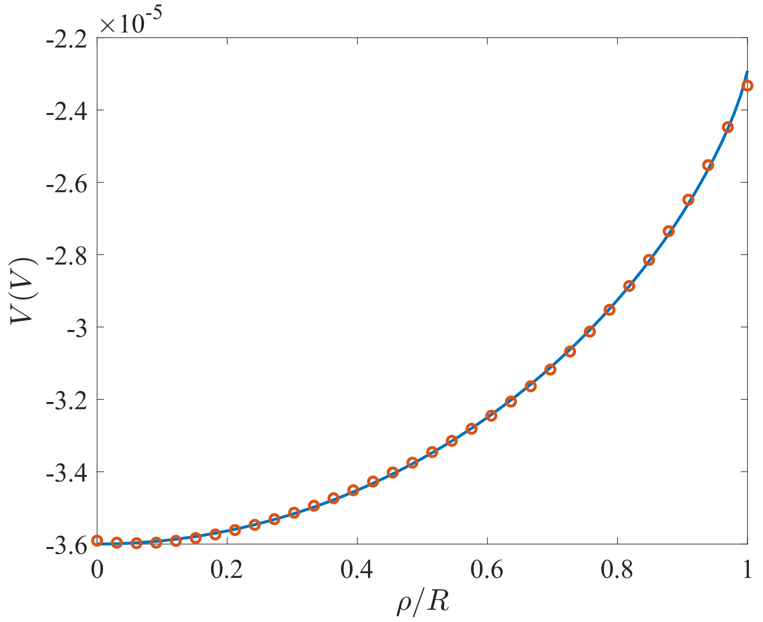

Fig. S2: Electric potential created by the background uniformly distributed negative density of charge σ, as a function of the distance ρ to the center of the circle. The exact value is drawn in blue solid line and our estimation, employing a 4-th order polynomial function to determine $V(\vec{r})$, with red circles.

Supporting information S3.- Monte Carlo principles enabling that the system reaches a final equilibrium state.

The principles and procedure used to let the system in Figure S1b) to evolve proceeds according to the following principles:

We consider two different configurations of the mobile ions, $\{\vec{r}_1, \vec{r}_2, \dots, \vec{r}_N\}$ and $\{\vec{r}_1', \vec{r}_2', \dots, \vec{r}_N'\}$, and introduce $w(\{\vec{r}_1, \vec{r}_2, \dots, \vec{r}_N\} \to \{\vec{r}_1', \vec{r}_2', \dots, \vec{r}_N'\})$ as the probability for the transition from $\{\vec{r}_1, \vec{r}_2, \dots, \vec{r}_N\}$ to $\{\vec{r}_1', \vec{r}_2', \dots, \vec{r}_N'\}$ in one step. Provided that:

i) the following condition holds.

$$w(\{\vec{r}_1, \vec{r}_2, \dots, \vec{r}_N\} \to \{\vec{r}_1', \vec{r}_2', \dots, \vec{r}_N'\})e^{-\beta U(\vec{r}_1, \vec{r}_2, \dots, \vec{r}_N)} = w(\{\vec{r}_1', \vec{r}_2', \dots, \vec{r}_N'\} \to \{\vec{r}_1, \vec{r}_2, \dots, \vec{r}_N\})e^{-\beta U(\vec{r}_1', \vec{r}_2', \dots, \vec{r}_N')}, \quad \text{(S1)}$$

which is known as detailed balance, and

ii) two arbitrary configurations can be connected to a certain chain of transitions, which is known as ergodicity, the equilibrium distribution is reached in the long-time limit, i.e. after a large enough number of steps [R1]. This is the basis of the Monte Carlo chain methods that are extensively used in the simulation of mesoscopic systems [R2].

Here, we implement an effective Monte Carlo dynamics for our system, specifically a variant of the so-called Metropolis algorithm. In each step of the dynamics, a mobile positive ion, the i-th one, is randomly chosen from the set of N ions, together with a random displacement $\vec{a}$ thereof. The following transition, from the "old" to the "new" configuration, is attempted:

$$\{\overrightarrow{r_1}, \dots, \vec{r_i}, \dots, \overrightarrow{r_N}\} \rightarrow \{\overrightarrow{r_1}, \dots, \vec{r_i} + \vec{a}, \dots, \overrightarrow{r_N}\} \qquad \text{(S2)}$$

The change of internal energy for the attempted transition is thus

$$\Delta U \equiv U_{new} - U_{old} = U(\overrightarrow{r_1}, \dots, \vec{r_i} + \vec{a}, \dots, \overrightarrow{r_N}) - U(\overrightarrow{r_1}, \dots, \vec{r_i}, \dots, \overrightarrow{r_N}). \quad \text{(S3)}$$

The typical *Metropolis* algorithm assigns the attempted transition a probability $p = \min\{1, e^{-\beta\Delta U}\}$, i.e. the transition is always accepted if it decreases U, whereas it is accepted with probability $e^{-\beta\Delta U} < 1$ if it increases U. This *Metropolis* rule can be formulated according to the relation $w\,(\{\overrightarrow{r_1}, \dots, \vec{r_i}, \dots, \overrightarrow{r_N}\} \to \{\overrightarrow{r_1}, \dots, \vec{r_i} + \vec{a}, \dots, \overrightarrow{r_N}\}) = \min\{1, e^{-\beta\Delta U}\}$, which verifies the balance condition in Eq. S1, and thus drives the system to equilibrium. Still, in

order to be more realistic, we also introduce an energy cost E_0 if the positive ion moves to an adjacent cell as a consequence of the attempted transition, i.e., when $\vec{r_i} \to \vec{r_i} + \vec{a}$. This accounts for an energy barrier hindering the motion of positive ions to a different cell. Therefore, we consider that

$$w\left(\{\vec{r_1}, \dots, \vec{r_i}, \dots, \vec{r_N}\} \to \{\vec{r_1}, \dots, \vec{r_i} + \vec{a}, \dots, \vec{r_N}\}\right) = e^{-\beta E_0 \delta(a,i)} \min\left\{1, e^{-\beta \Delta U}\right\}, \text{(S4)}$$

in which $\delta\,(a, i) = 1$ if $\vec{r_i}$ and $\vec{r_i} + \vec{a}$ belong to different cells, whereas $\delta\,(a, i) = 0$ if $\vec{r_i}$ and $\vec{r_i} + \vec{a}$ belong to the same cell. As compared with the case $E_0 = 0$, the transitions that involve crossing a cell boundary are slowed down by the Arrhenius-like factor $e^{-\beta E_0}$. Since the reverse transition also involves crossing a cell boundary, the balance condition in Eq. S1 is preserved and this more realistic dynamics also drives the system to equilibrium.

Using our Monte Carlo algorithm, the system transits through different ion configurations, jumping from one to another with the probability just described above. In this way, we construct a *Markov* chain with detailed balance, and thus the final—for very long times—state of the system corresponds to equilibrium at the bath temperature T, described by the canonical distribution, which minimizes the free energy. For low temperatures, the system would reach the state with minimal energy (or, at least, a local minimum), since the entropic contribution to the free energy becomes negligible. This is the equilibrium state resulting from the accumulation of extra negative charge on the surface of seeds during the plasma treatment.

[R1] V. Kampen, N. Godfried. Stochastic Processes In Physics and Chemistry. 3rd ed. Amsterdam: Elsevier, 2007 ISBN 9780080475363.

[R2] D. Frenkel, B. Smit. Understanding Molecular Simulation: From Algorithms to Applications. 3rd ed. Elsevier, 2023 ISBN 9780323902922.

Supporting information S4.- SEM and EDX analysis of barley seeds.

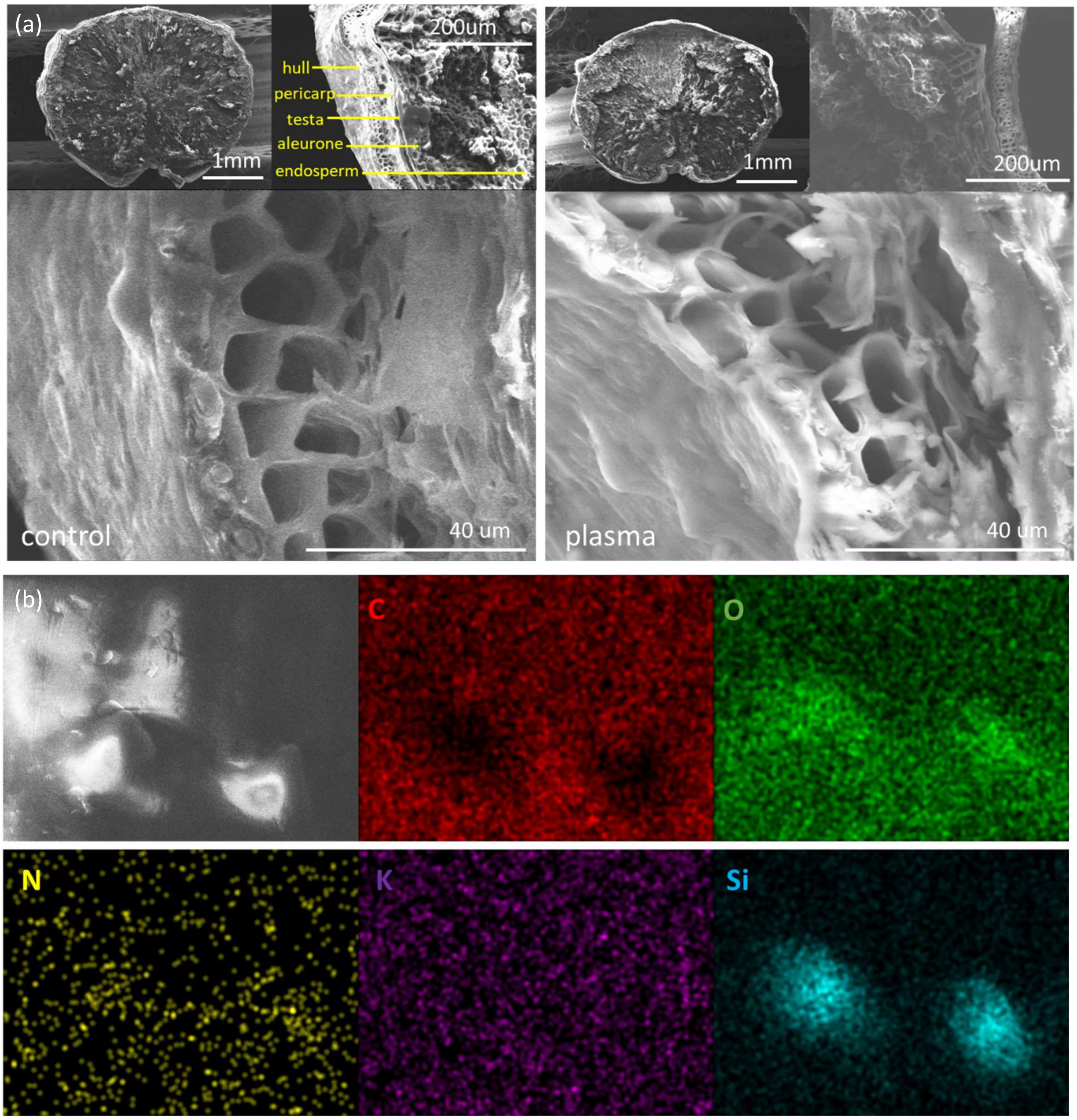

Fig. S4. (a) SEM images of cuts of the barley seeds highlighting the internal structure of the pericarp, indicating the main identified layers (yellow color). No significant differences in morphology for length scales of several microns are observed between the control and the plasma treated seeds. (b) SEM image and EDX analysis of the surface of the seed in the form of C, O, N, K and Si maps, corresponding to a barley seed after a plasma treatment for 3min. The presence of SiO_2 agglomerates on the barley seed surface is clearly appreciated in these images

Supporting information S5.- Additional EDX analysis of barley seeds cuts

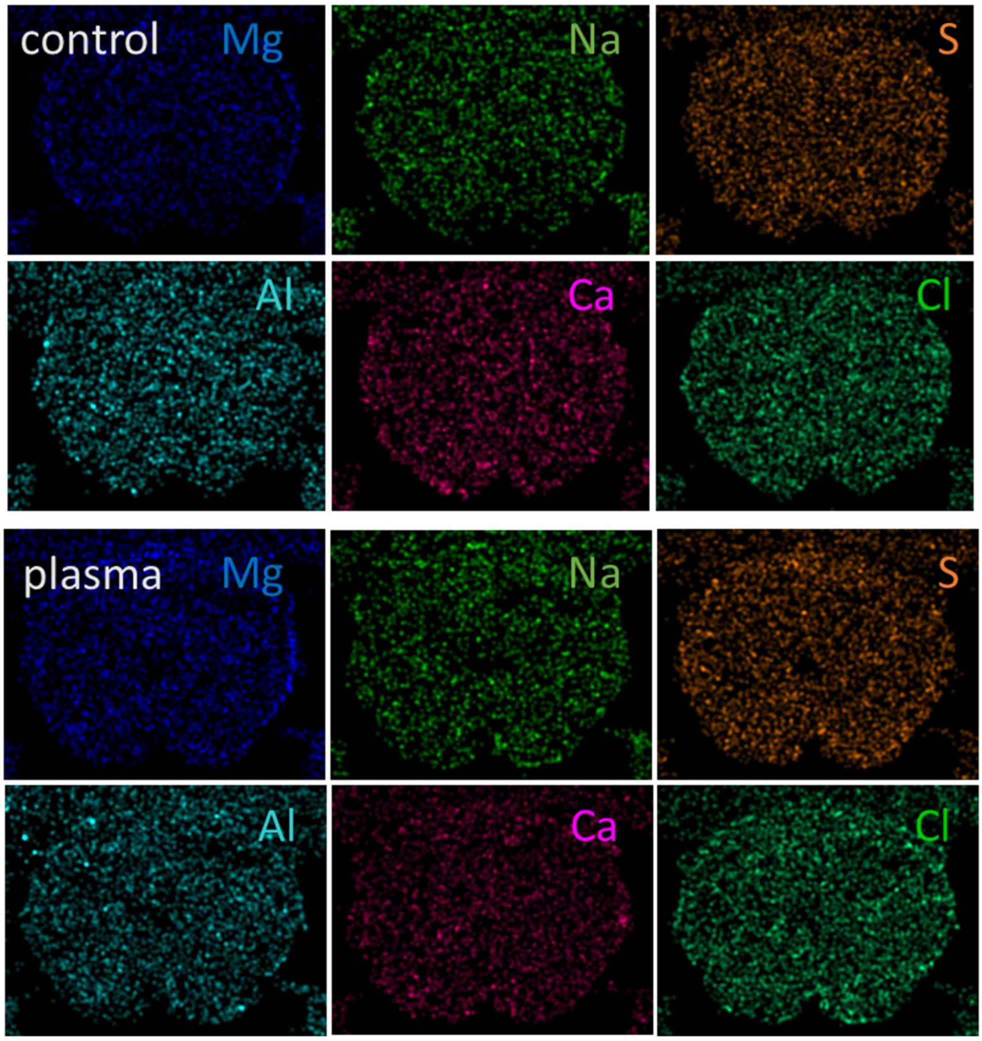

Fig. S5. EDX additional maps of the seed cuts before and after the plasma treatment corresponding to the minority elements detected: Mg, Na, S, Al, Ca, and Cl.

Supporting information S6.- Etching profiles according to protocol 2 for barley seeds before and after plasma treatment.

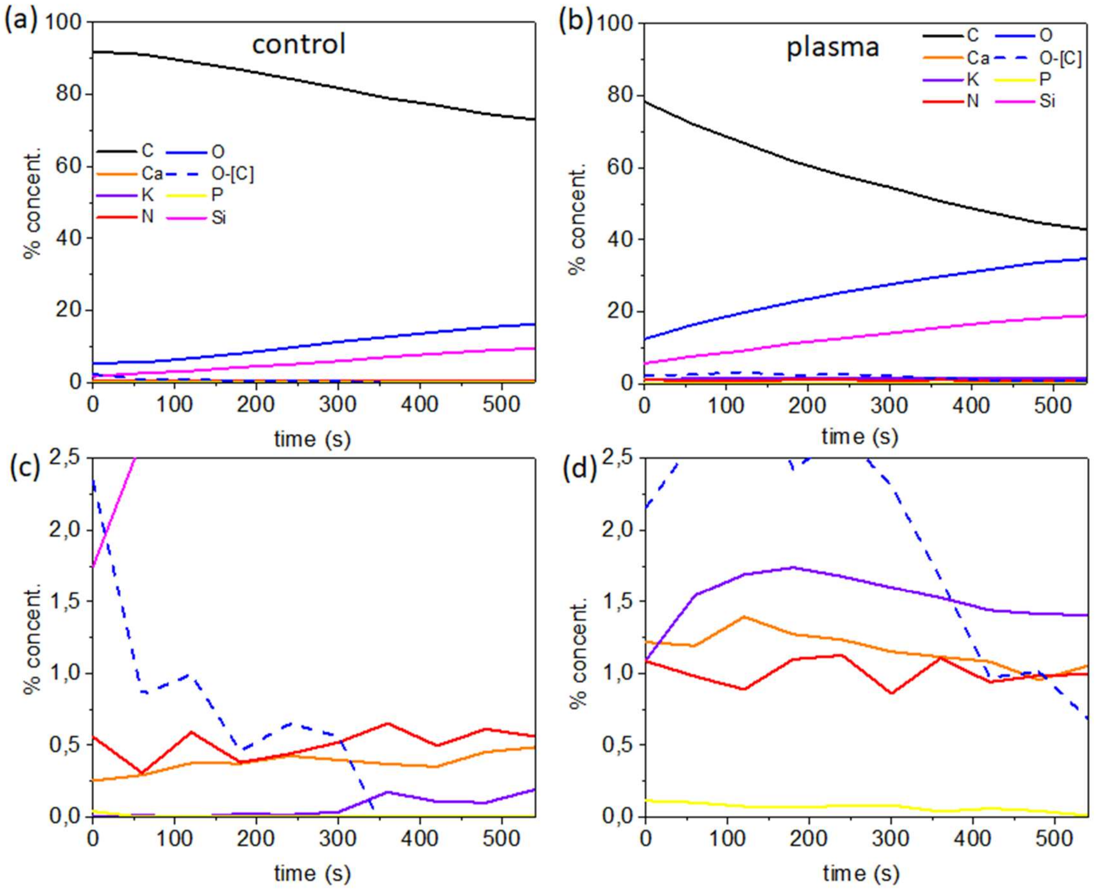

Fig. S6. Element depth profiles according to protocol 2 for the reference (a, c) and plasma treated (b,d) seeds. Dashed lines represent the depth profile of oxygen associated to the organic component of the outer layers of seeds (i.e., once discounting the oxygen associated to silicon). These depth profiles confirm that the enrichment in N, P and K after the plasma treatment extends to a depth of at least 200 nm.

Supporting information S7.- XPS spectra of minority elements recorded after GCIB depth profiling of pristine and plasma treated barley seeds.

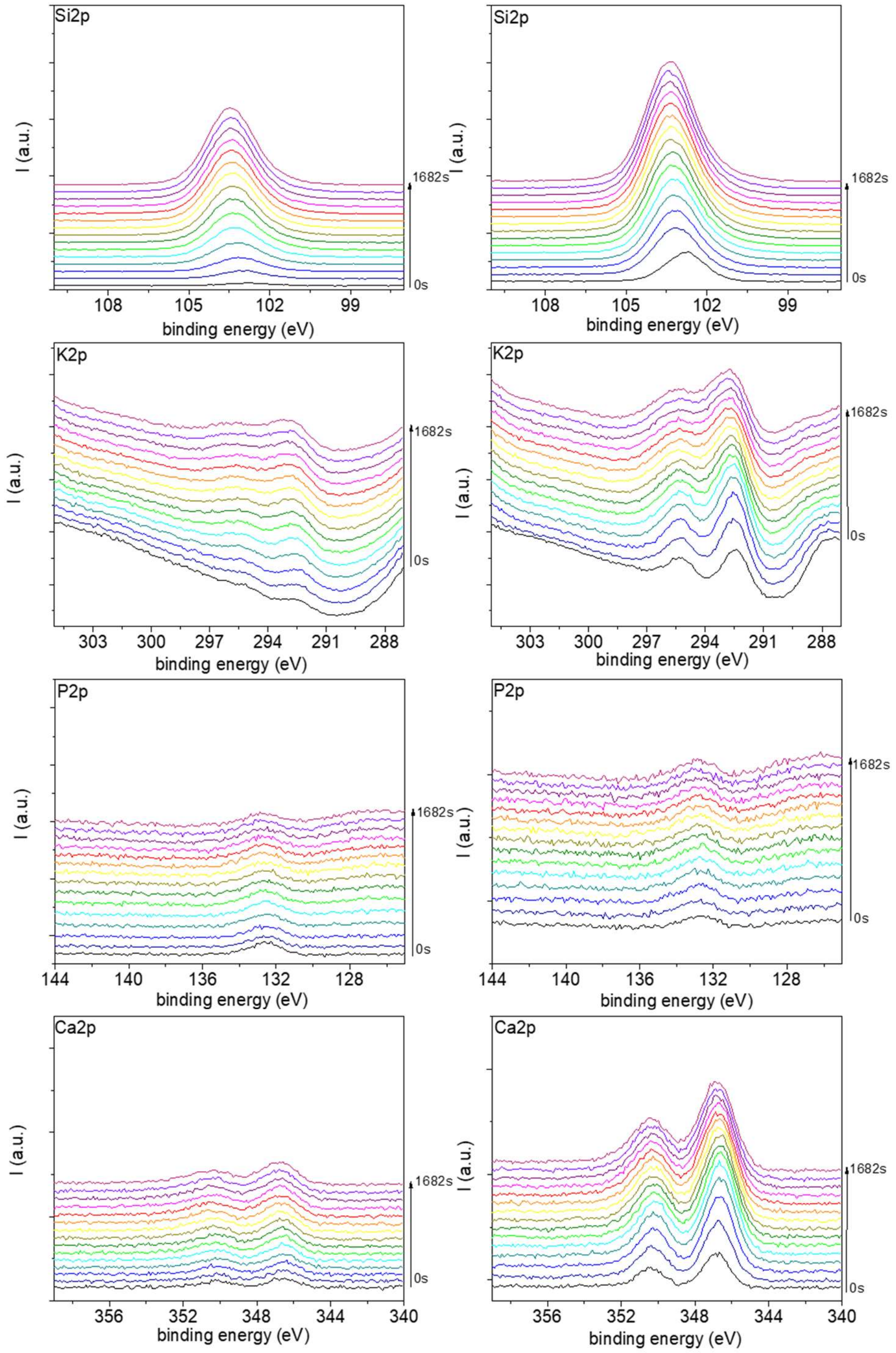

Fig. S7. Selected Si 2p, K 2p, P 2p and Ca 2p spectra taken after GCIB depth profiling (according to the etching time indicated with the arrow) for control (a) and plasma treated (b) barley seeds.

Supporting information S8.- Summary of the binding energies and atomic concentration of the functional groups at the barley surface before and after the plasma treatment obtained by fitting the XPS spectra

XPS functional group	Binding energy (eV)	Fitted At. Concentration (%)	
		Untreated	Plasma treated
-C-C	284.5	66.1	56.1
-C-O	286.0	27.8	33.3
-C=O	287.5	9.1	10.6
C-O	532.7	11.4	91.8
C=O	533.2	88.1	7.7
N-O	536.2	0.5	0.5
Si-C-O	102.8	100	73.4
Si-O	103.4	0	26.6
K-Cl/-SO_4	292.8	100	100
Ca-O	346.7	100	100
P-O	132.7	100	100

Supporting information S9.- Evolution of the internal energy per ion.

Fig. S9 showcases the evolution of the internal energy U/N per (positive mobile) ion U as a function of the number of algorithm iterations. This plot is not a time evolution, but a representation of the Monte Carlo dynamics that, following the energy premises in eqs. 3-7 in the main text is designed to drive the system towards equilibrium. The plot shows that, on average, the energy decreases from the value in the initial state configuration to the final state, becoming roughly constant after 6x10^5 steps (although there are thermal fluctuations in the final state, these fluctuations do not alter the basic tendency revealed by the Monte Carlo dynamics).

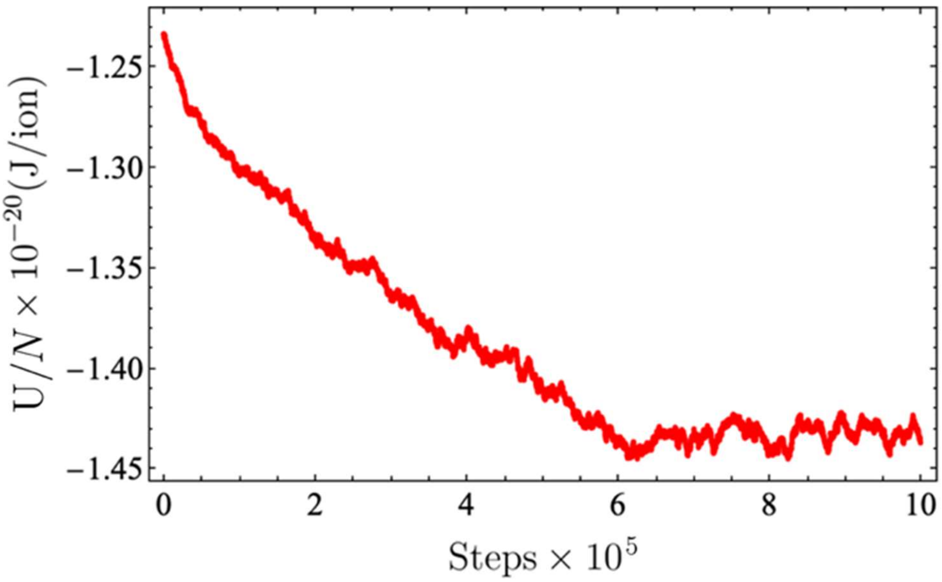

Fig. S9. Evolution of the internal energy of the positive ions in the Monte Carlo dynamics. The internal energy is plotted as a function of the number of steps in the algorithm. The energy decreases with the number of steps, until it reaches, on average, a constant value for a number of steps $> 6 \times 10^5$, corresponding to the equilibrium state.

Supporting information S10.- Calculation of the distribution of positive charge in the final state of the system.

The following procedure was used to evaluate the distribution of positive charge in the final state (c.f., Figure 7b in the main text) as a function of the radial coordinate ρ. Taking the numerical density n as the number of ions per unit area, $N(\rho, \rho + \Delta\rho)$ will represent the number of mobile positive ions in an annulus of radii ρ and $\rho + \Delta\rho$. Then

$$N(\rho, \rho + \Delta\rho) = n(\rho)\pi[(\rho + \Delta\rho)^2 - \rho^2] \simeq n(\rho)2\pi\rho\Delta\rho \quad \text{(S5)}$$

To improve the statistics for $n(\rho)$, it has been averaged over all configurations of positive ion distributions calculated from $\text{Step} = 6 \times 10^5$ to $\text{Step} = 10^6$, i.e., once the equilibrium state has been reached.
